# The Untapped Potential of Virtual Reality in Rehabilitation of Balance and Gait in Neurological Disorders

**DOI:** 10.3389/frvir.2021.641650

**Published:** 2021-03-11

**Authors:** Emily A. Keshner, Anouk Lamontagne

**Affiliations:** 1 Department of Health and Rehabilitation Sciences, Temple University, Philadelphia, PA, United States; 2 School of Physical and Occupational Therapy, McGill University, Montreal, QC, Canada; 3 Virtual Reality and Mobility Laboratory, CISSS Laval—Jewish Rehabilitation Hospital Site of the Centre for Interdisciplinary Research in Rehabilitation of Greater Montreal, Laval, QC, Canada

**Keywords:** posture, locomotion, sensorimotor, avatar, intervention

## Abstract

Dynamic systems theory transformed our understanding of motor control by recognizing the continual interaction between the organism and the environment. Movement could no longer be visualized simply as a response to a pattern of stimuli or as a demonstration of prior intent; movement is context dependent and is continuously reshaped by the ongoing dynamics of the world around us. Virtual reality is one methodological variable that allows us to control and manipulate that environmental context. A large body of literature exists to support the impact of visual flow, visual conditions, and visual perception on the planning and execution of movement. In rehabilitative practice, however, this technology has been employed mostly as a tool for motivation and enjoyment of physical exercise. The opportunity to modulate motor behavior through the parameters of the virtual world is often ignored in practice. In this article we present the results of experiments from our laboratories and from others demonstrating that presenting particular characteristics of the virtual world through different sensory modalities will modify balance and locomotor behavior. We will discuss how movement in the virtual world opens a window into the motor planning processes and informs us about the relative weighting of visual and somatosensory signals. Finally, we discuss how these findings should influence future treatment design.

## INTRODUCTION

Virtual reality (VR) is a compelling and motivating tool that can be used to modulate neural behavior for rehabilitation purposes. Virtual environments can be developed as simple two-dimensional visual experiences and as more complex three-dimensional gaming and functional environments that can be integrated with haptics, electromyography, electroencephalography, and fMRI. These environments can then be used to address a vital need for rehabilitative training strategies that improve functional abilities and real-world interaction. There has been a concerted effort to determine whether motor learning in VR transfers to the physical world (Levac et al., 2019). Although this is important for determining measurable goals for intervention with VR, the sole focus on diminishing a motor deficit without controlling the perceptual factors within the virtual environment could actually interfere with task transfer and the rehabilitation process. Mounting evidence suggests that VR contributes to the complex integration of information from multiple sensory pathways and incorporates the executive processing needed to perceive this multimodal information (Keshner and Fung, 2019). Thus, VR is a rehabilitation tool that can be designed to address the perception-action system required for motor planning, a vital part of motor learning and performance, as well as motor execution.

In humans, common neural activation during action observation and execution has been well documented. A variety of functional neuroimaging studies, using fMRI, positron emission tomography, and magnetoencephalography, have demonstrated that a motor resonance mechanism in the premotor and posterior parietal cortices occurs when participants observe or produce goal directed actions (Grèzes et al., 2003; Hamzei et al., 2003; Ernst and Bülthoff, 2004). Mirror neurons in the ventral premotor and parietal cortices of the macaque monkey that fire both when it carries out a goal-directed action and when it observes the same action performed by another individual also provides neurophysiological evidence for a direct matching between action perception and action production (Rizzolatti and Craighero, 2004).

The concept of perception-action coupling has been accepted since Gibson (Gibson, 1979) who argued that when a performer moves relative to the environment, a pattern of optical flow is generated that can then be used to regulate the forces applied to control successive movements (Warren, 1990). In other words, we organize the parameters of our movement in relation to our perception of the signals we are receiving from the environment, and the change resulting from our action will then change the environment we must perceive for any subsequent action. Thus, how we perceive the environmental information will always affect how we organize and execute an action. Not taking into account the environmental factors that influence perception during training may well confound any assessments of performance and transfer of training (Gorman et al., 2013).

The essence of VR is the creation of the environment. Environments are created for many purposes ranging from industrial to entertainment and gaming to medical (Rizzo and Kim, 2005; Levin et al., 2015; Garrett et al., 2018; Keshner et al., 2019). Environments have been developed to overlay virtual objects on the physical world (i.e., augmented reality) or to present a fully artificial digital environment (i.e., VR). Rarely, however, is the motor ability of the performer considered in the design of these environments. In this study we will present work from our laboratories in which we specifically focused on coupling of the environmental and motion parameters.

## MANIPULATING VISUAL MOTION INFORMATION (OPTIC FLOW)

In a seminal paper initially published in 1958, Gibson formulated the foundations of what would become an influential theory on the visual control of locomotion (Gibson, 2009). Among key aspects of this theory was the role visual kinaesthesis, or optic flow, in the perception of egomotion and control of locomotion (Warren, 2009). Since early 2000, VR technology has undoubtedly contributed to our understanding of the role of optic flow and other sources of visual information in the control of human posture and locomotion (Warren et al., 2001; Wilkie and Wann, 2003).

Several psychophysical phenomena are attributed to the impact of optic flow on perception. Presence and immersion describe the user’s belief in the reality of the environment (Slater, 2003). These terms have been used interchangeably, but they should be distinguished from the perspective of the measurement tool. According to Slater (Slater, 2003), immersion is a measure of the objective level of sensory fidelity provided by a VR system; presence is a measure of the subjective psychological response of a user experiencing that VR system.

Vection is the sensation of body motion in space produced purely by visual stimulation. This illusory motion of the whole body or of body parts is induced in stationary observers viewing environmental motion (Dichgans and Brandt, 1972; Dichgans et al., 1972; Palmisano et al., 2015). Examples of such a conflict occur in daily life when watching a moving train and sensing that it is the train and not yourself who is moving (Burr and Thompson, 2011). It is generally agreed that this illusion of self-motion results from a sensory conflict or mismatch that cannot be resolved by the CNS. Vection has also been defined more broadly as the conscious subjective experience of self-motion (Ash et al., 2013) that is crucial for successful navigation and the prevention of disorientation in the real world (Riecke et al., 2012).

Lastly, perception of self-motion is a challenging problem in the interpretation of multiple sensory inputs, requiring the neural combination of visual signals (e.g., optic flow), vestibular signals regarding head motion, and also somatosensory and proprioceptive cues (Deangelis and Angelaki, 2012). To perform successfully, we need to link sensory information to the context of the movement and determine whether there is a match between the visual motion and our vestibular and somatosensory afference and then shape our movement to accurately match the demands of the environment (Hedges et al., 2011). Consistent multisensory information about self-motion, rather than visual-only information, has been shown to reduce vection and improve both heading judgment and steering accuracy (Telford et al., 1995). Subjects demonstrated no compensation for self-motion that was defined solely by vestibular cues, partial compensation (47%) for visually defined self-motion, and significantly greater compensation (58%) during combined visual and vestibular self-motion (Dokka et al., 2015). Body posture will orient to a visual, somatosensory, or vestibular reference frame depending on the task, behavioral goals, and individual preference (Streepey et al., 2007b; Lambrey and Berthoz, 2007). Development across the lifespan and damage to the CNS may produce a shift in sensory preferences and thereby alter the responsiveness to any of the sensory pathways resulting in altered motor behavior (Slaboda et al., 2009; Yu et al., 2020). Thus, understanding how virtual environment parameters influence motor planning and execution is essential if we are to use virtual reality effectively for training and intervention.

### Evidence From VR-Based Neuroimaging Studies

Through the combination of VR and neuroimaging tools, key brain regions involved in the perception and use of optic flow during simulated “locomotor tasks” were unveiled. Human motion area hMT+ and ventral intraparietal cortex (VIP) play a role in the perception of egomotion from optic flow (Morrone et al., 2000; Dukelow et al., 2001; Wall and Smith, 2008), while a region of the intraparietal sulcus (IPS) would be responsible for identifying heading from optic flow information (Peuskens et al., 2001; Liu et al., 2013). PET and MRI studies indicate that when both retinal and vestibular inputs are processed, there are changes in the medial parieto-occipital visual area and parietoinsular vestibular cortex (Brandt et al., 1998; Dieterich and Brandt, 2000; Brandt et al., 2002), as well as cerebellar nodulus (Xerri et al., 1988; Kleinschmidt et al., 2002), suggesting a deactivation of the structures processing object-motion when there is a perception of physical motion. When performing VR-based steering tasks, additional regions such as the premotor cortex and posterior cerebellum get recruited (Field et al., 2007; Billington et al., 2010; Liu et al., 2013). The latter two brain regions would contribute to the planning and online monitoring of observer’s perceived position in space, while also contributing to the generation of appropriate motor responses (Field et al., 2007; Liu et al., 2013). Interestingly, a study which combined EEG to a VR setup during Lokomat-supported locomotion also showed an enhancement in premotor cortex activation when performing a steering task in first or third person view compared to conditions where no locomotor adaptations were required, which the authors also attributed to an enhanced need for motor planning (Wagner et al., 2014).

In most recent VR-based neuroimaging studies, individuals are immersed in more realistic environments and perform tasks of increasing complexity such as attending to or avoiding moving objects during simulated self-motion, where both perceived self-motion and object motion are at play (Calabro and Vaina, 2012; Huang et al., 2015; Pitzalis et al., 2020). Collectively, the fundamental knowledge acquired through VR-based neuroimaging experiments is key as it has allowed rehabilitation scientists to pose hypotheses and explain impaired locomotor behaviors and the heterogeneity of thereof in clinical populations with brain disorders such as stroke or Parkinson’s disease. Existing VR-based neuroimaging studies, however, remain foremost limited by their lack of integration of actual locomotor movements and nonvisual self-motion cues (Chaplin and Margrie, 2020). Multisensory convergence takes place at multiple levels within the brain. As an example, animal research has shown that MSTd and the parietoinsular vestibular contribute to a coherent percept of heading by responding both to vestibular cues and optic flow (Duffy, 1998; Angelaki et al., 2011)—an observation that was made possible by exposing the animal to a combination of optic flow manipulation and actual body translation in space. In human research, the emergence of mobile neuroimaging tools (e.g., fNIRS, EEG) and more robust analysis algorithms now makes it possible to examine the neural substrates of actual locomotion (Gramann et al., 2011; Brantley et al., 2018; Gennaro and De Bruin, 2018; Nordin et al., 2019; Wagner et al., 2019). Studies combining VR as well as other technologies (e.g., motion platform, robotic devices) to mobile neuroimaging can be expected, in the near future, to flourish and advance our understanding of locomotor control in complex, comprehensive yet controlled multisensory environments.

### What Have We Learned From Lab-Based Postural Control Studies

**FIGURE 1 |:**
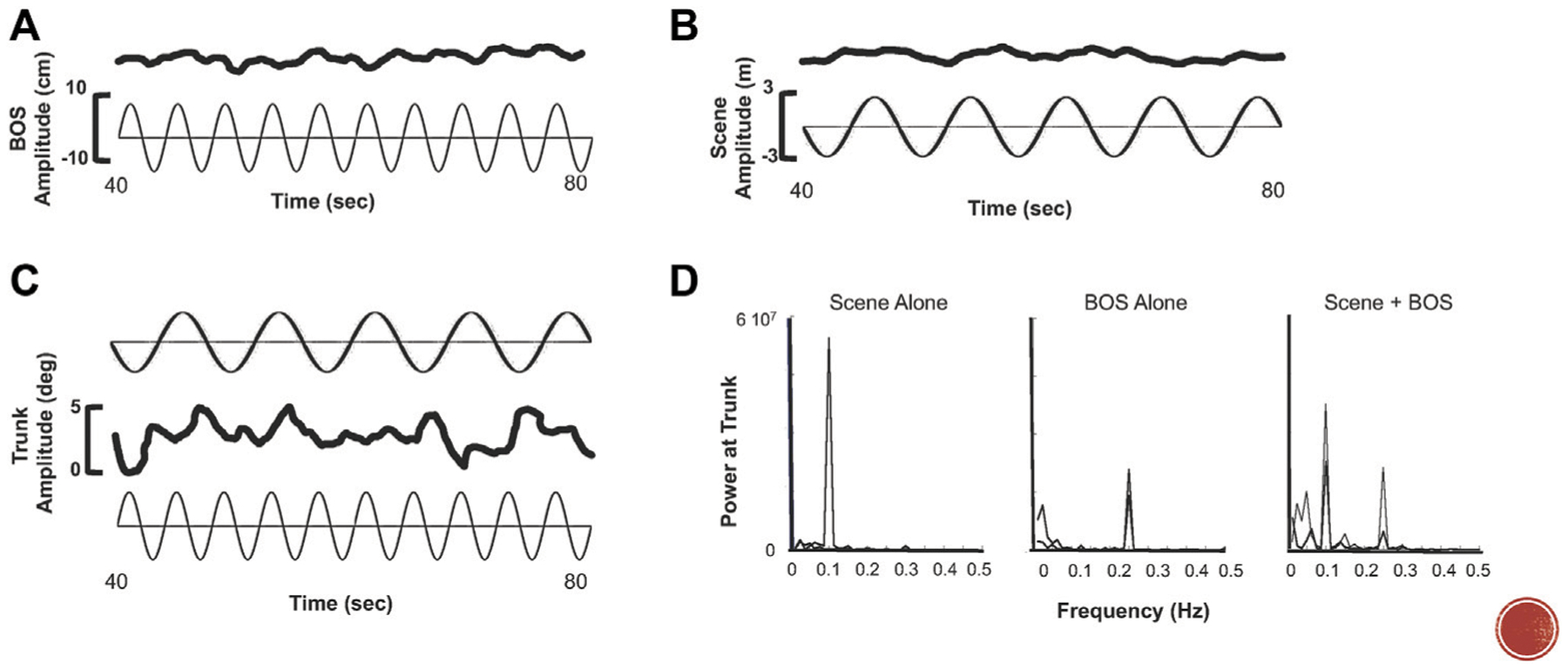
**(A)** Trunk excursion (top trace) to sinusoidal a-p translation (bottom trace) of the base of support (BOS) at 0.25 Hz. **(B)** Trunk excursion (top trace) to sinusoidal a-p optic flow (scene) at 0.1 Hz. **(C)** Trunk excursion (middle trace) when 0.25 Hz motion of the BOS (bottom trace) and 0.1 Hz of the scene (top trace) occur simultaneously. **(D)** FFT analysis demonstrating power at the trunk reflects frequency of the stimulus, i.e., the scene **(left)**, the BOS **(middle)**, and simultaneous BOS and scene motion **(right)**.

Our studies in immersive VR environments (using both projection and head mounted display (HMD) technology) reveal that it is nearly impossible for a performer to ignore the dynamic visual stimulus (Keshner and Kenyon, 2000, 2009; Cleworth et al., 2012). As shown in a seminal paper by Dichgans et al. (1972), sensitivity to a virtual visual stimulus is greatly increased when there is a combination of meaningful inputs (Dichgans et al., 1972). Measures of head, trunk, and lower limb excursions revealed that the majority of participants compensated in the opposite direction but at the same frequency for motion of a translating platform in the dark (Keshner et al., 2004). When on a stationary platform with a translating visual scene, participants matched the frequency and direction of the scene motion with their head and trunk but at much smaller amplitudes. Combining platform and visual scene motion produced the greatest amplitudes of motion occurred in all body segments. Additionally, frequency content of that movement reflected both the frequencies of the platform and the visual scene suggesting that the sense of presence was greatly intensified when producing self-motion within a dynamic visual environment (Figure 1).

**FIGURE 2 |:**
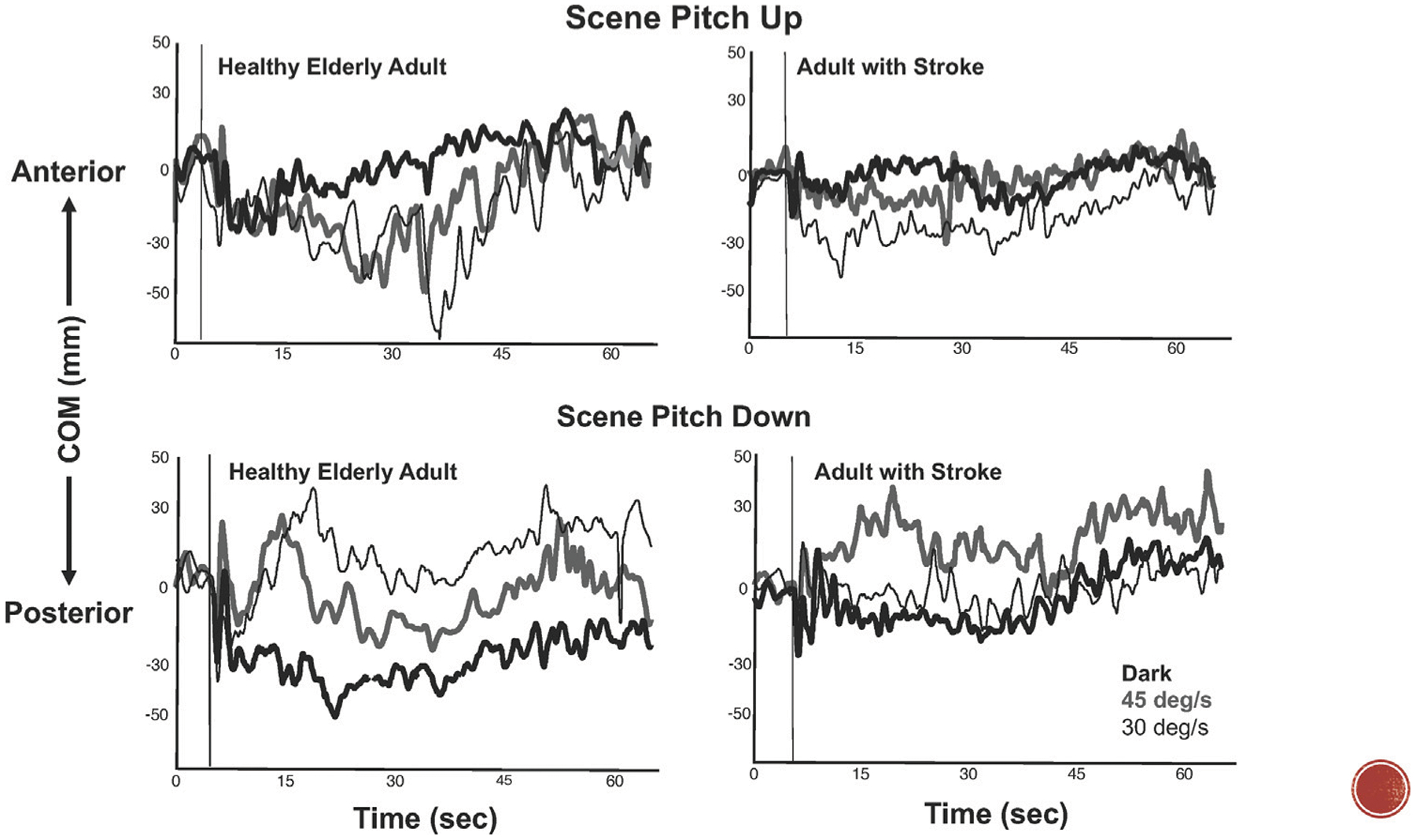
Center of mass (COM) excursions during a-p translations of a platform at 0.25 Hz while standing in the dark (bold black line) and while viewing continuous pitch rotations of optic flow at 30 deg/sec (thin black line) and 45 deg/sec (bold gray line). ***Top graphs:*** responses to pitch-up rotations of the scene in a healthy 62-year-old adult **(left)** and 65 year-old-adult with right hemiplegia **(right)**. ***Bottom graphs:*** responses to pitch down rotations of the scene in a healthy elderly adult **(left)** and elderly adult with stroke **(right)**. Vertical thin line indicates start of optic flow field.

These results suggest that the postural response was modulated by all of the available sensory signals. In fact, the data strongly establish that kinematic variables of postural behavior are responsive to the metrics of the multimodal inputs. In particular, postural behavior has been shown to be influenced by the velocity, direction, and frequency parameters of the optic flow (Figure 2). For example, healthy young adults standing on a tilting platform in a 3-wall projection environment (Dokka et al., 2010; Wang et al., 2010) modified the direction, velocity, and amplitude of their COM motion in relation to the velocity of a visual scene rotating in the pitch direction. When standing on a stable surface, healthy young adults matched the direction of their head and trunk swaying to the direction of visual motion in both pitch and roll.

Although velocity and direction may be governed by optic flow, magnitude of the response does vary across individuals (Keshner et al., 2004; Streepey et al., 2007a; Dokka et al., 2009). Healthy young adults in front of a wide field of view virtual scene that translated in the anterior-posterior (a-p) direction stood upon a rod that supported 100% or 45% of their foot length; thus, the base of support was whole or narrowed. Even in these healthy, young adults, success at maintaining a vertical orientation was compromised when standing on the narrowed base of support; however, the sway of about half the participants matched the frequency of the visual scene whereas the other half did not demonstrate a predominant frequency. This suggests a preferential sensory referencing in some participants to the sinusoidal visual signals and in others to the proprioceptive signals from the body. Intraindividual variability and task dependency that is demonstrated in the virtual environment (Keshner and Kenyon, 2000; Streepey et al., 2007b) imply that postural control is both task and organism dependent and should not be treated as a stereotypical, automatic behavior.

A developmental impact on the ability to process optic flow was revealed during a functional sit-to-stand task (Slaboda et al., 2009). Healthy children (8–12 years) and adults (21–49 years) were seated in a virtual environment that rotated in the pitch and roll directions. Participants were told to stand either (1) concurrent with onset of visual motion or (2) after an immersion period in the moving visual environment and (3) without visual input. Both adults and children reduced head and trunk angular velocity after immersion in the moving visual environment. Unlike adults, children demonstrated significant differences in displacement of the head center of mass during the immersion and concurrent trials when compared to trials without visual input. These data support previous reports (Keshner and Kenyon, 2000; Keshner et al., 2004) of a time-dependent effect of vision on response kinematics in adults. Responses in children are more influenced by the initial presence or absence of vision from which we might infer poorer error correction in the course of an action.

### Utilizing Optic Flow for Postural Rehabilitation

Optic flow in the virtual environment robustly influences the organization of postural kinematics. This influence, however, fluctuates with the integrity of the CNS and the perceptual experiences of each individual. Sensory signals are often reweighted in individuals as they age and with neurological disability, which then alters the postural response to optic flow (Slaboda et al., 2009; Yu et al., 2018). Thus, the success of any therapeutic intervention employing VR needs to consider the parameters of visual motion of the virtual environment. There are, however, some global precepts that can guide the deployment of any VR intervention. Specifically, studies have consistently demonstrated that (1) the direction of full-field optic flow will regulate the direction of postural sway (Keshner and Kenyon, 2009); (2) increasing velocity will increase the magnitude of postural sway (Dokka et al., 2009; Wang et al., 2010); (3) multiple sensory frequencies will be reflected in the body segment response frequencies (Keshner et al., 2004; Slaboda et al., 2011a); and (4) the influence of optic flow becomes more substantial during self-motion (Dokka et al., 2010).

Training individuals that have instability and sensory avoidance to produce effective postural behaviors have obvious value and there are some studies demonstrating carryover to the functional postural behavior of individuals with labyrinthine loss (Haran and Keshner, 2008; Bao et al., 2019), Parkinson’s disease (Bryant et al., 2016; Nero et al., 2019; Rennie et al., 2020), and stroke (Van Nes et al., 2006; Madhavan et al., 2019; Saunders et al., 2020). The very strong directional effect of optic flow on posture and spatial orientation (Keshner and Kenyon, 2000) would support incorporating this technology into any balance rehabilitation program.

The ability to change response magnitudes relative to visual velocity has been demonstrated in young healthy adults and in individuals diagnosed with dizziness (Keshner et al., 2007), stroke (Slaboda and Keshner, 2012), and cerebral palsy (Yu et al., 2018; Yu et al., 2020) when support surface tilts were combined with sudden rotations of the visual field. Both of these variables are time dependent and require further clinical trials to determine appropriate dosage of these interventions. Sensory reweighting, however, has been shown to be frequency dependent and requires control of multimodal stimuli. Angular displacements of the head, trunk, and head with respect to the trunk consistently revealed that healthy individuals linked their response parameters to visual inputs and those with visual sensitivity as measured with a Rod and Frame test could not use the visual information to appropriately modulate their responses. Instead, individuals with visual dependence, with or without a history of labyrinthine dysfunction, tended to produce longer duration and larger magnitude angular velocities of the head than healthy individuals in all planes of motion and at all scene velocities (Keshner and Dhaher, 2008; Wright et al., 2013).

These findings could be explained by an inability to adapt the system to the altered gains resulting from the neurological damage so that they could not accommodate to sensory signals with which they had no prior experience (i.e., constant motion of the visual world). A similar outcome was observed in healthy young adults who received vibrotactile noise on the plantar surface of the foot during quiet stance. Stochastic resonant vibration of the lower limbs in older adults and patients with stroke has been shown to reduce postural instability (Van Nes et al., 2004; Guo et al., 2015; Lu et al., 2015; Leplaideur et al., 2016). Although vibration does not shorten the time to react to instability, it can decrease the amplitude of fluctuation between the controlled body segment and unstable surface thereby increasing the likelihood that a corrective response will be effective. While viewing visual field rotations, however, magnitude and noise of their center of mass (COM) and center of pressure (COP) responses increased rather than decreased with vibration (Keshner et al., 2011) suggesting that, by increasing noise in the system, individuals were unable to fully compensate for the disturbances. The use of noise and sensory mismatch to encourage desensitization or compensation is currently being explored for the treatment of dizziness and postural instability (Pavlou et al., 2011; Pavlou et al., 2012; Sienko et al., 2017; Bao et al., 2019). Individuals with dizziness from concussion or labyrinthine dysfunction have also been exposed to erroneous or conflicting visual cues (visual-vestibular mismatch) while attempting to maintain balance (Bronstein and Pavlou, 2013; Pavlou et al., 2013). Results suggest that exposure to unpredictable and noisy environments can be a valuable tool for motor rehabilitation. Dosages (e.g., timeframe and range of stimulation) of the intervention need to be further explored with controlled trials.

### What Have We Learned From Lab-Based Locomotor Studies

An extensive body of literature has examined the role of visual self-motion in the control of locomotion by selectively manipulating the direction or speed of the optic flow provided through the virtual environment. Our work and that of others have shown that one’s walking speed is affected by changing optic flow speeds and show an out-of-phase modulation pattern. In other words, slower walking speeds are adopted at faster optic flow speeds while faster walking speeds are observed at slower optic flows (Pailhous et al., 1990; Konczak, 1994; Prokop et al., 1997; Varraine et al., 2002). Such strategy would allow reducing the incongruity that arises from the mismatch between proprioceptive information from the legs and the visual flow presented in the virtual simulation (Prokop et al., 1997; Lamontagne et al., 2007). The presence of optic flow during treadmill walking also influences one’s ability to correct small stepping fluctuations (Salinas et al., 2017). Compelling evidence also support the role of optic flow in the control of locomotor steering (Jahn et al., 2001; Warren et al., 2001; Mulavara et al., 2005; Turano et al., 2005; Bruggeman et al., 2007). In the latter body of literature, a shift in the focus of expansion of the optic flow is externally induced and this causes the participants to perceive a shift in their heading direction. As a result, the participants correct the perceived shift by altering their walking trajectory in the opposite direction. Our team has also shown that depending on whether the shift in the focus of expansion is induced through rotational vs. translational flow, different steering strategies emerge (Sarre et al., 2008). In the former scenario, a steering strategy characterized by head, trunk, and foot reorientation is observed, while the latter scenario rather induces a typical “crab walk pattern” characterized by a change of walking trajectory with very little body segment reorientation. Such crab walking pattern has also been reported in other VR studies that used translational optic flow (Warren et al., 2001; Berard et al., 2009).

Interestingly, if the same rotational optic flow is generated via a simulated head yaw rotation (camera rotation in VR) vs. an actual head rotation, a different locomotor behavior also emerges, whereby the simulated but not the actual head rotation results in a trajectory deviation (Hanna et al., 2017). Such findings support the potential contribution of the motor command (here neck and oculomotor muscles) in heading estimation (Banks et al., 1996; Crowell et al., 1998). These findings also corroborate the presence of multisensory integration of both visual and nonvisual information (e.g., vestibular, proprioceptive, and somatosensory) to generate a single representation of self-motion and orientation in space (De Winkel et al., 2015; Acerbi et al., 2018).

### Influences of Optic Flow on Locomotor Rehabilitation

Collectively, the above-mentioned observations demonstrate that while locomotor adaptions rely on multisensory integration, vision and here, more specifically, optic flow exert a powerful influence on the observed behavior. Findings presented also provide concrete examples as to how optic flow information can be selectively manipulated to alter locomotor behavior. Thus, not only is the replication of reality in VR not a necessity, but the selective manipulation of the sensory environment can and should as needed be capitalized on to promote the desired outcome. To allow for such manipulations to be effective in a given clinical population, however, the latter must show a residual capacity to perceive and utilize optic flow information while walking.

The perception of optic flow and its use in locomotion have been examined in several clinical populations such as older adults (Chou et al., 2009; Lalonde-Parsi and Lamontagne, 2015) and Parkinson’s disease patients (Schubert et al., 2005; Davidsdottir et al., 2008; Young et al., 2010; Van Der Hoorn et al., 2012), but let us use stroke as an example to demonstrate applications in rehabilitation. Following stroke, the perception of optic flow often is preserved (Vaina et al., 2010; Ogourtsova et al., 2018) but becomes affected when the lesion is located in rostrodorsal parietal and occipitoparietal areas of the brain, which are involved in global motion perception (Vaina et al., 2010). In presence of unilateral spatial neglect (USN), the bilateral perception of optic flow (e.g., optic flow direction and coherence) becomes dramatically altered (Ogourtsova et al., 2018). In fact, altered optic flow perception along with USN severity as measured by clinical tests explain 58% of the variance in locomotor heading errors in individuals with poststroke USN (Ogourtsova et al., 2018). Such observations emphasize the need to consider the role of visual-perceptual disorders in poststroke locomotor impairments.

Beyond studies examining the perception of optic flow perception, our group has also examined the use of optic flow during locomotion by manipulating the direction or speed of the virtual environment (Lamontagne et al., 2007; Lamontagne et al., 2010; Berard et al., 2012; Aburub and Lamontagne, 2013). From these experiments emerged three main observations: (1) globally, the ability to utilize OF information during walking is altered following stroke; (2) there is however a large heterogeneity across individuals, ranging from no alterations to profound alterations in locomotor responses to optic flow manipulations; and (3) most individuals show some degree of modulation (albeit incomplete or imperfect) of their locomotor behavior in response to optic flow manipulation. Thus, one can infer that there is potential to induce the desired locomotor adaptations through optic flow manipulation in stroke survivors. However, integration of such manipulations in intervention studies for locomotor rehabilitation is scare and evidence of effectiveness is lacking.

In 2012, Khang and collaborators combined treadmill training to optic flow speed manipulation for 4 weeks and examined the effects on balance and locomotion following stroke (Kang et al., 2012). Unfortunately, although the study showed larger posttraining gains in walking speed and endurance in the optic flow manipulation group vs. control groups receiving either conventional treadmill training or a stretching program, the study design did not allow to dissociate the contribution of VR itself from that of the optic flow manipulation. Furthermore, it is unclear if any online walking speed adaptation took place during training given the absence of a self-pace mode on the treadmill. A study from Bastian’s lab also showed that combining split-belt walking to an incongruent optic flow that predicted the belt speed of the next step enhanced the rate of learning during split-belt locomotor adaptations in healthy individuals (Finley et al., 2014). To date, however, the integration of such paradigm as part of an intervention to enhance poststroke gait asymmetry remains to be examined.

## INTERACTION WITH AVATARS

In recent years, and thanks to technological development that allows tracking and displaying body movements in real-time in a virtual environment, the development of avatar-based paradigms in rehabilitation has emerged. Unlike virtual humans or agents which are controlled by computer algorithms, avatars are controlled by the users and “mimic” their movements in real-time. The avatar can represent either selected body parts (e.g., arms or legs) or the full body. They can also be viewed from a first-person perspective (1 PP) or third-person perspective (3 PP). In the paragraphs below, we are mainly concerned with exploring the impact of avatar-based feedback as a paradigm to enhance postural control and locomotion in clinical populations, but literature on upper extremity research that explores mechanisms is also examined.

### Why Avatar-Based Feedback

Potential principles of action of avatar-based feedback are multiple and, as stated in a recent expert review on virtual reality, they open a “plethora of possibilities for rehabilitation” (Tieri et al., 2018). When exposed to virtual simulations representing body parts or the full body, a phenomenon referred to as virtual embodiment can develop. This sense of embodiment translates as the observer experiencing a sense of owning the virtual body simulation (ownership) and of being responsible for its movement (agency) (Longo et al., 2008; Pavone et al., 2016). While such sense of embodiment is subjectively reported as higher for 1 PP vs. 3 PP (Slater et al., 2010; Petkova et al., 2011; Pavone et al., 2016), we argue that the latter perspective remains very useful for postural and locomotor rehabilitation (as one does not necessarily look down at their feet, for instance, when standing or walking). The similarity between the virtual vs. real body part(s) (Tieri et al., 2017; Kim et al., 2020; Pyasik et al., 2020), the real-time attribute or synchrony of the simulation with actual movements (Slater et al., 2009; Kim et al., 2020), and the combination of sensory modalities (e.g., visuotactile (Slater et al., 2008) or visuovestibular (Lopez et al., 2008; Lopez et al., 2012)) are factors that enhance the illusory sensation.

Neuroimaging experiments indicate that the premotor areas (pre-SMA and BA6) are involved in the sense of agency (Tsakiris et al., 2010), while ownership would be mediated through multimodal integration that involves multiple brain areas including the somatosensory cortex, intraparietal cortex, and the ventral portion of the premotor cortex (Blanke, 2012; Guterstam et al., 2019; Serino, 2019). Mirror neurons located in the ventral premotor cortex and parietal areas, but also in other regions such as visual cortex, cerebellum, and regions of the limbic system, also fire when an individual observes someone else’s action (Molenberghs et al., 2012) and are likely activated when exposed to avatar-based feedback. Passively observing modified (erroneous) avatar-based feedback also leads to activation of brain regions associated with error monitoring (Pavone et al., 2016; Spinelli et al., 2018), which is a process essential for motor learning.

During actual locomotion, the performance of a steering task while exposed to avatar feedback provided in 1 PP or 3 PP was shown to induce larger activation in premotor and parietal areas compared to movement-unrelated feedback or mirror feedback (Wagner et al., 2014). While such enhanced activation appears primarily caused by the motor planning and visuomotor demands associated with gait adaptations (Wagner et al., 2014), it may as well have been potentiated by a sense of embodiment and/or mirror neuron activations. More recently, another study reported an event-related synchronization in central-frontal (likely SMA) and parietal areas both during actual and imagined walking while exposed to 1 PP avatar-based feedback (Alchalabi et al., 2019). This event-related synchronization was attributed by the authors to the high sense of agency experienced during these conditions. Together, the latter two locomotor studies provide preliminary evidence that the body of knowledge on avatar-based feedback gathered primarily via upper extremity experiments can be extended, at least in part, to locomotion. Most importantly, observations from neuroimaging experiments as a whole indicate that avatar-based feedback does modulate brain activation. Through repeated exposure, such a paradigm could thus support neuronal reorganization and recovery following a neurological insult.

From a more pragmatic perspective, avatar-based feedback also capitalizes on the remarkable ability of the human brain to perceive and interpret biological motion information (Johansson, 1973). This remarkable ability allows recognizing features such as the nature of the activity being performed (e.g., walking), gender and emotion, even when exposed to impoverished visual simulations such as point-light displays (Johansson, 1973; Troje, 2002; Atkinson et al., 2004; Schouten et al., 2010). For similar reasons, we as human can easily identify even the most subtle limp when observing a walking pattern, which makes avatar-based feedback a potentially powerful approach to give and receive feedback on complex tasks such as locomotion. Avatar feedback further allows providing real-time feedback on the quality of movement (knowledge of performance) (Liu et al., 2020b), which is especially challenging for clinicians to do. In line with previous literature on embodiment presented earlier, avatar-based feedback may also impact recovery by enhancing movement awareness, which is affected in clinical populations such as stroke (Wutzke et al., 2013; Wutzke et al., 2015).

### Manipulation of Avatar-Based Feedback

Avatar-based feedback can be manipulated in different ways (e.g., view, available sensory modality, modified vs. unmodified feedback, etc.), yet the optimal parameters to obtain the desired responses remain unclear. In a recent study from our laboratory, we posed the question “which avatar view between the front, back and side view, yields the best instantaneous improvement in poststroke gait asymmetry?” (Liu et al., 2020b). Participants were tested while exposed to 3 PP full-body avatars presented either in the front, back, or paretic side view and resulting changes in gait symmetry were examined. The side view, which likely provides the best perspective on the temporal-distance parameters of gait, was the only view that induced enhanced spatial symmetry but only in those participants who initially presented a larger step on the paretic side. This finding was caused by the participants increasing their step length on the nonparetic side when exposed to the avatar, which resulted in improved symmetry only in those with a large paretic step. Such an observation suggests that the initial profile of the participant matters and, by extension, that avatar-based feedback may not be suitable for all individuals. Of note, manipulating 3 PP viewing angle of a virtual arm was also found to alter kinematic outcomes during a reaching task performed while standing (Ustinova et al., 2010). Avatar view thus emerges as a factor to consider in the design of an intervention.

**FIGURE 3 |:**
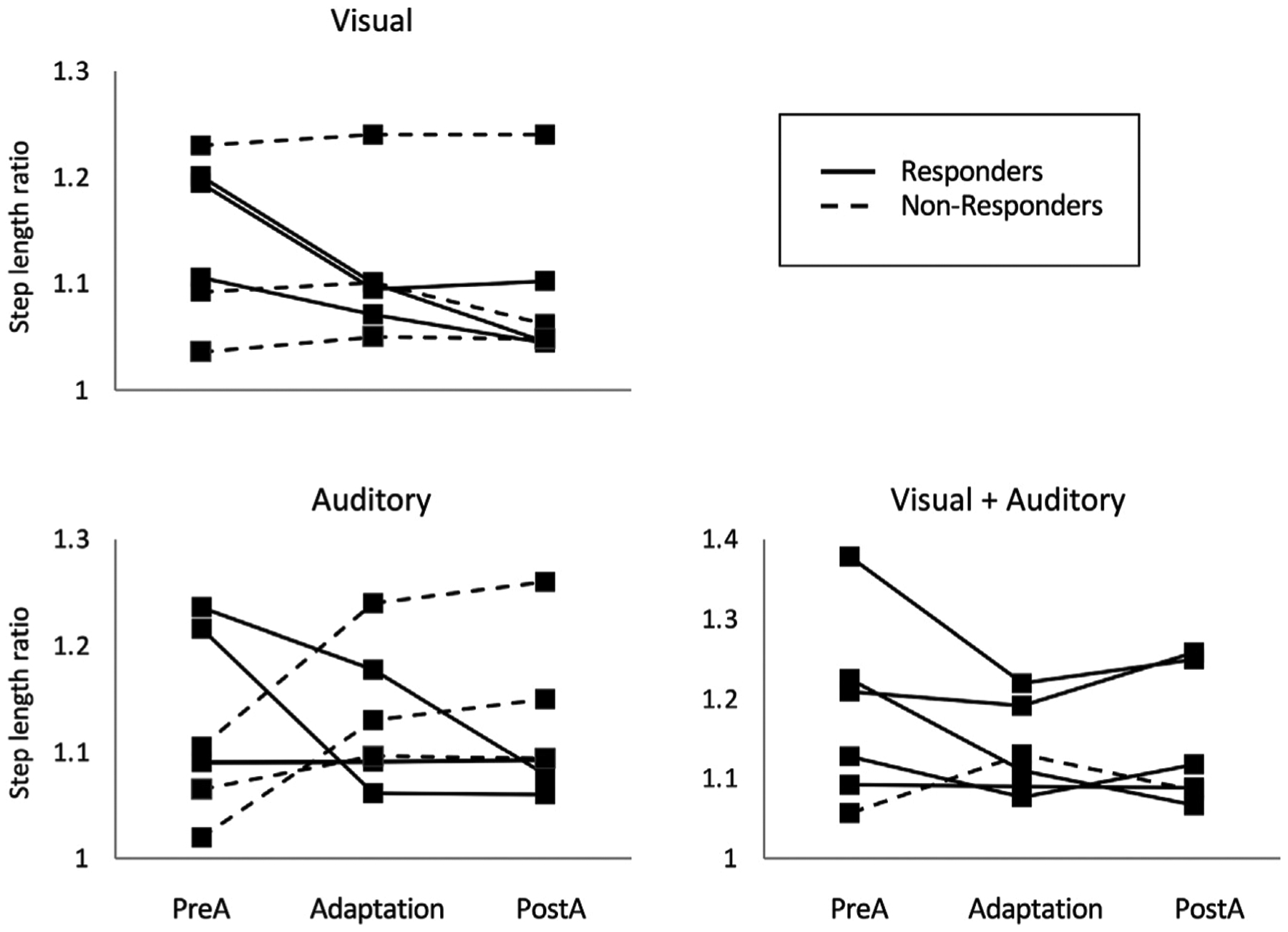
Step length ratio values exhibited by stroke survivors walking on a self-paced treadmill while exposed to avatar-based feedback in the visual, auditory, and combined (visual + auditory) sensory modality. Values are presented for the preadaptation (no avatar for 30 s), adaptation (avatar present for 1 min), and postadaptation periods (avatar removed for 1 min). Responders, that is individuals showing a reduction of their step length ratio during the adaptation period, are represented by a plain line, while non-responders are represented by a dotted line. Note the larger number of responders to the combined vs. individual sensory modalities.

In a second series of experiments, we examined the impact of modulating the sensory modality of avatar-based feedback on poststroke gait asymmetry. The feedback consisted either of a 3 PP visual avatar in the side view (visual), footstep sounds (auditory), or a combination of visual avatar and footstep sounds (combined modality) (Liu et al., 2020a). Although these results are preliminary, there is a clear implication that combining sensory modalities yielded the largest improvements in spatial symmetry (Figure 3). These results are in agreement with prior studies on other types of multimodal simulation, such as the combination of a visual avatar to tactile or haptic feedback, that were found to have additional beneficial effects on the performance of healthy individuals performing a stepping task (Koritnik et al., 2010) and on the ability of individuals with spinal cord injury to integrate virtual legs to their body representation (Shokur et al., 2016).

The evidence supports the use of multimodal feedback to modulate or train functional locomotion from a rehabilitation perspective. In upper extremity rehabilitation research, a well-studied approach consists of artificially increasing the perceived performance error through visual or haptic feedback (i.e., error augmentation paradigm) (Israely and Carmeli, 2016; Liu et al., 2018). Similarly, manipulating avatar-based feedback offers an opportunity to modify the locomotor behavior. In 2013, Kannape and Blanke manipulated the temporal delay of avatar-based feedback and found that, while gait agency decreased with longer delays, participants “systematically modulated their stride time as a function of the temporal delay of the visual feedback”, making faster steps in presence of incongruous temporal feedback (Kannape and Blanke, 2013). More recently, a preliminary study examined the impact of stride length manipulation through hip angle modifications and found a clear trend toward larger step lengths when exposed to larger avatar step lengths (Willaert et al., 2020). Such experiments provide preliminary evidence that modified avatar-based feedback can lead to locomotor adaptations either in the temporal or spatial domain. Avatar-based feedback can further be augmented with visual biofeedback on specific kinematic or kinetic features of the gait cycle. In children with cerebral palsy, for instance, avatar-based feedback was augmented with biofeedback on knee or hip excursion, as well as step length, resulting in further improvements in those parameters compared to avatar-based feedback alone (Booth et al., 2019).

Collectively, findings in this section demonstrate that avatar-based feedback can be effectively manipulated to modify locomotor behavior and target specific features of gait. It can also be used as a mean to enhance the control of movement through brain computer interface (Wang et al., 2012; King et al., 2013; Nierula et al., 2019). Further research is needed, however, to understand how it can be optimized to promote the desired outcome. At this point in time, intervention studies that specifically focus on repeated exposure to avatar-based feedback as an intervention for postural or locomotor rehabilitation in populations with sensorimotor disorders are crucially lacking.

## INTERACTION WITH VIRTUAL HUMANS

### External Cuing

Inclusion of external agents (i.e., virtual humans) in virtual scenarios has emerged as a means to modulate locomotion in the context of rehabilitation. Such an approach stems in part from a large body on research on the use of external sensory cueing (e.g., visual or auditory) to modulate the temporal-distance factors of gait both in healthy individuals (Rhea et al., 2014; Terrier, 2016) and individuals with gait disorders (Roerdink et al., 2007; Spaulding et al., 2013). It also stems from the fact that when two individuals walk together (i.e., when exposed to biological sensory cues), the locomotor behavior is modulated as a result of a mutual interaction between the two walkers (Ducourant et al., 2005) and a phenomenon of “gait synchronization”, whereby a follower matches the gait pattern of the leader, can be observed (Zivotofsky and Hausdorff, 2007; Zivotofsky et al., 2012; Marmelat et al., 2014; Rio et al., 2014). Such gait synchronization can be fostered through different sensory channels (e.g., visual, tactile, and auditory) and is enhanced with multimodal simulations (Zivotofsky et al., 2012). In postural tasks, a similar phenomenon of synchronization of postural sway is observed when individuals are standing and having a physical contact (Reynolds and Osler, 2014), while looking at each other (Okazaki et al., 2015) or while sharing a cooperative verbal task (Shockley et al., 2003). Given the flexibility and control afforded by VR, virtual humans can also be used to “cue” and modulate behavior, as demonstrated through different studies which have examined instantaneous effects on locomotion (Meerhoff et al., 2017; Meerhoff et al., 2019; Koilias et al., 2020). While promising as a tool for rehabilitation, however, evidence of effectiveness of external cueing through virtual humans as an intervention either for posture or locomotion remains to be established.

### Pedestrian Interactions

Virtual humans can also be used for the assessment and training of complex locomotor tasks such as avoiding collisions with other pedestrians, which is a task essential for independent community walking (Patla and Shumway-Cook, 1999; Shumway-Cook et al., 2003). Collision avoidance heavily relies on the sense of vision, in comparison to other senses such as audition (Souza Silva et al., 2018). For this reason, most of the literature has focused on the visual modality to infer the control variables involved (Cutting et al., 1995; Gerin-Lajoie et al., 2008; Olivier et al., 2012; Fajen, 2013; Darekar et al., 2018; Pfaff and Cinelli, 2018). VR has brought major contributions to our understanding of collision avoidance, with some elements that are especially relevant to rehabilitation. A first key element is that different collision avoidance strategies emerge when avoiding virtual objects vs. virtual humans. The latter were shown to lead to smaller obstacle clearances which were interpreted as a use of less conservative avoidance strategies (Lynch et al., 2018; Souza Silva et al., 2018). Factors that may explain such difference include the level of familiarity with the task (i.e., avoiding pedestrians is far more common than avoiding an approaching cylinder/sphere), the social attributes of the virtual humans (Souza Silva et al., 2018), as well as the local motion cues arising from the limb movements that were shown to shape some aspects of the avoidance strategy (Lynch et al., 2018; Fiset et al., 2020). A combination of real-world and VR studies has also shown that the collision avoidance strategy in response to a human interferer is modulated by factors such as the static vs. moving nature of the interferer (Basili et al., 2013) as well as its direction (Huber et al., 2014; Knorr et al., 2016; Buhler and Lamontagne, 2018; Souza Silva et al., 2018) and speed of approach (Huber et al., 2014; Knorr et al., 2016). All these factors can easily and effectively be manipulated in VR to promote the desired behavior and expose users to the diversity of scenarios they would encounter while walking in the community. Whether personal attributes of the interferers impact on collision avoidance strategies, however, is still unclear (e.g., Knorr et al., 2016; Bourgaize et al., 2020) and deserves further investigations.

VR-based studies on pedestrian interactions and collision avoidance, including recent work from our laboratory, have proven to be useful in unveiling the altered collision avoidance strategies experienced by several populations such as healthy older adults (Souza Silva et al., 2019; Souza Silva et al., 2020), individuals with mild traumatic brain injury (Robitaille et al., 2017), and individuals with stroke with (Aravind and Lamontagne, 2014; Aravind et al., 2015; Aravind and Lamontagne, 2017a; b) and without USN (Darekar et al., 2017b; a). We and others have also shown that simultaneously performing a cognitive task alters the collision avoidance behavior and can compromise safety by generating addition collisions (Aravind and Lamontagne, 2017a; Robitaille et al., 2017; Lamontagne et al., 2019a; Souza Silva et al., 2020; Deblock-Bellamy et al., 2021—accepted). In parallel to those clinical investigations, other studies carried out in healthy individuals have demonstrated that similar obstacle avoidance strategies are used when avoiding virtual vs. physical humans, although with subtle differences in walking speed and obstacle clearance (Sanz et al., 2015; Buhler and Lamontagne, 2018; Olivier et al., 2018; Bühler and Lamontagne, 2019). Such results support the use of virtual humans as a valid approach to evaluate and train pedestrian interactions as experienced in daily life. Pedestrian interactions can be facilitated by the use of omnidirectional treadmills that allow speed and trajectory changes (Lamontagne et al., 2019b; Soni and Lamontagne, 2020) and should be added as an essential dimension of community walking to complement existing VR-based interventions that focus on locomotor adaptations (e.g., Yang et al., 2008; Mirelman et al., 2011; Mirelman et al., 2016; Peruzzi et al., 2017; Richards et al., 2018).

## DISCUSSION

A recent review (Tieri et al., 2018) of the contributions of VR to cognitive and motor rehabilitation suggests that the most promising effects of VR are the ability to multitask in a virtual environment that can replicate the demands of a physical environment, i.e., it is an ecologically valid rehabilitation tool. Our data and others indicate that the sensory environment can be effectively manipulated to promote a desired motor outcome so that engagement with the task is encouraged and the process of active motor control is facilitated even if the VR environment deviates from physical reality. In order to accomplish this, however, we need to understand the properties of VR technology that create meaningful task constraints such as sensory conflict and error augmentation. One of the greatest weaknesses afflicting identification of the value of VR to rehabilitation is the application of the term “VR” to describe a myriad of paradigms that do not meet the requirements to truly be considered virtual reality. In order for a VR guided rehabilitation program to be successful, immersion in an environment that produces presence and embodiment is necessary if the user is to respond in a realistic way (Kenyon et al., 2004; Keshner and Kenyon, 2009; Tieri et al., 2018). Thus, only by activating the perception-action pathways for motor behavior will appropriate emotional reactions, incentives to act, and enhanced performance take place.

Results from the studies presented here clearly demonstrate that one of the primary contributions of VR to physical rehabilitation interventions is the ability to engage the whole person in the processes of motor learning and control (Sveistrup, 2004; Adamovich et al., 2009). Principal strengths of utilizing VR for rehabilitation is that it encourages motor learning through practice and repetition without inducing the boredom often resulting during conventional exercise programs. With this technology, interventions can be designed to address the particular needs of each individual, activity can be induced through observation, and intensity of practice can be modified in response to individual needs. But, in order to accomplish any of these goals, it is essential that the clinicians understand how and why they are choosing VR to meet their treatment goals and how to optimally tailor treatments for a desired outcome. Factors to consider when choosing to incorporate VR into a treatment intervention include whether (1) the donning of devices such as goggles alter motor performance (Almajid et al., 2020); (2) the manipulation of objects in the environment will alter the sense of presence; (3) certain populations are more susceptible to the virtual environment and, therefore, will respond differently than predicted (Slaboda et al., 2011b; Almajid and Keshner, 2019); and (4) a visual or multimodal presentation of the environment and task will be best to obtain the desired behavior. In addition, significant weaknesses remain in our understanding about the impact of VR on physical rehabilitation because of the dearth of well-designed clinical trials that consider dosages and technological equivalencies (Weiss et al., 2014).

In this article, we have focused on research demonstrating how multisensory signals delivered within a virtual environment will modify locomotor and postural control mechanisms. Studies using motor learning principles and complex models of sensorimotor control demonstrate that all sensory systems are involved in a complex integration of information from multiple sensory pathways. This more sophisticated understanding of sensory processing and its impact on the multisegmental body has altered our understanding of the causality and treatment of instability during functional movements. Therefore, incorporating VR and other applied technologies such as robotics has become essential to supplying the impact of multisensory processing on motor control (Saleh et al., 2017).

Motivation and enjoyment are an essential component in a rehabilitation program, and we are in no way suggesting that computer gaming and exercise and augmented reality technologies should be ignored because they do not necessarily deliver all components of a virtual reality environment. Rather, we are contending that there are additional pathways for training and modifying postural and locomotor behaviors in an immersive and multimodal virtual environment that will facilitate transfer of training of the neurophysiological and musculoskeletal mechanisms underlying functional motor behavior.
